# Multidimensional frequency domain analysis of full-volume fMRI reveals significant effects of age, gender, and mental illness on the spatiotemporal organization of resting-state brain activity

**DOI:** 10.3389/fnins.2015.00203

**Published:** 2015-06-16

**Authors:** Robyn L. Miller, Erik B. Erhardt, Oktay Agcaoglu, Elena A. Allen, Andrew M. Michael, Jessica A. Turner, Juan Bustillo, Judith M. Ford, Daniel H. Mathalon, Theo G. M. Van Erp, Steven Potkin, Adrian Preda, Godfrey Pearlson, Vince D. Calhoun

**Affiliations:** ^1^The Mind Research NetworkAlbuquerque, NM, USA; ^2^Department of Mathematics and Statistics, University of New MexicoAlbuquerque, NM, USA; ^3^Department of Electrical and Computer Engineering, University of New MexicoAlbuquerque, NM, USA; ^4^Bergen Center for Neuropsychiatric ResearchBergen, Norway; ^5^Geisinger Autism and Developmental Medicine InstituteLewisburg, PA, USA; ^6^Department of Psychology and Neuroscience, Georgia State UniversityAtlanta, GA, USA; ^7^Department of Psychiatry, University of New Mexico School of MedicineAlbuquerque, NM, USA; ^8^Department of Psychiatry, University of California San Francisco School of MedicineSan Francisco, CA, USA; ^9^Department of Psychiatry and Human Behavior, School of Medicine, University of California IrvineIrvine, CA, USA; ^10^Department of Psychiatry, Yale University School of MedicineNew Haven, CT, USA

**Keywords:** fMRI, spatiotemporal frequency domain, schizophrenia, multidimensional Fourier transform, brain dynamics

## Abstract

Clinical research employing functional magnetic resonance imaging (fMRI) is often conducted within the connectionist paradigm, focusing on patterns of connectivity between voxels, regions of interest (ROIs) or spatially distributed functional networks. Connectivity-based analyses are concerned with pairwise correlations of the temporal activation associated with restrictions of the whole-brain hemodynamic signal to locations of a priori interest. There is a more abstract question however that such spatially granular correlation-based approaches do not elucidate: Are the broad spatiotemporal organizing principles of brains in certain populations distinguishable from those of others? Global patterns (in space and time) of hemodynamic activation are rarely scrutinized for features that might characterize complex psychiatric conditions, aging effects or gender—among other variables of potential interest to researchers. We introduce a canonical, transparent technique for characterizing the role in overall brain activation of spatially scaled periodic patterns with given temporal recurrence rates. A core feature of our technique is the spatiotemporal spectral profile (STSP), a readily interpretable 2D reduction of the native four-dimensional brain × time frequency domain that is still “big enough” to capture important group differences in globally patterned brain activation. Its power to distinguish populations of interest is demonstrated on a large balanced multi-site resting fMRI dataset with nearly equal numbers of schizophrenia patients and healthy controls. Our analysis reveals striking differences in the spatiotemporal organization of brain activity that correlate with the presence of diagnosed schizophrenia, as well as with gender and age. To the best of our knowledge, this is the first demonstration that a 4D frequency domain analysis of full volume fMRI data exposes clinically or demographically relevant differences in resting-state brain function.

## Introduction

Much fMRI research focuses onestimates offunctional connectivity between fixed parcellations or weightings of voxel space (van den Heuvel and Pol, 2010; Erhardt et al., 2011a; Biswal, 2012; Calhoun and Adali, 2012; Smith, 2012). Moreover, with some notable and interesting exceptions (Cordes et al., 2001a; Chang and Glover, 2010; Su et al., 2013; Ciuciu et al., 2014; Sasai et al., 2014), an overwhelming proportion of “connectomic” studies remain focused primarily on the evidence of functional connectivity provided by measurements of linear correlation between network timecourses. While a correlation-driven network connectivity framework is optimal for certain questions, the brain is operating on many scales simultaneously and we can miss useful information or even bypass interesting questions by structuring so much analysis around the assumptions that:

1. Temporal behavior is relevant primarily through its correlative properties.
2. Popular methods of collapsing space to a small number signal-carrying nodes generally produce networks that preserve temporal variability at the most salient spatial scales.
3. Spatiotemporal properties of information flow through inter-node tissue, i.e., through the often substantial gray matter spatial complement of the ROIs or networks under explicit consideration, can be safely ignored.

Here we investigate the relative contributions of 3D spatial intensity patterns of roughly homogeneous directional periodlengths (from small to large) moving at different temporal frequencies through the 4D fMRI signal. The Fourier transform was chosen because it is canonical, powerful, and transparently interpretable. In the present investigation it also proves entirely sufficient to expose significant group differences in spatiotemporal hemodynamic activation patterns.

**Figure 1:**
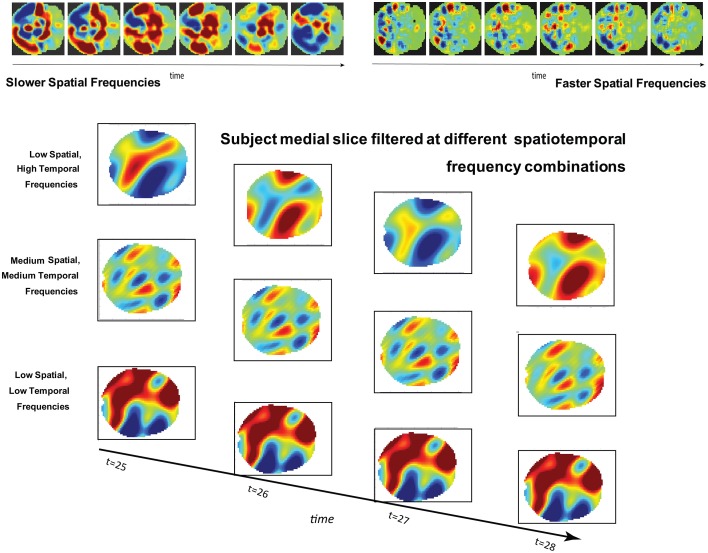
**(Top):** Simulated brain slice dynamics with random assignment of frequencies and phases to larger (left) and smaller (right) subregions of the slice. The active regions have similar behavior in both cases, but this behavior plays out on differently scaled spatial patterns in each. **(Bottom)**: Four consecutive timeframes of one subject's medial slice data filtered at indicated combinations of spatial and temporal frequencies.

The present work demonstrates that, in the case of resting-state fMRI data, treating the whole brain in time as a single 4D signal exposes significant group-level distinctions in general spatiotemporal patterning of hemodynamic activation (Figure 1). While correlational network analysis is a large and growing presence in fMRI research (Bullmore and Sporns, 2009; Friston, 2011; Friston and Price, 2011; Shirer et al., 2012; Smith, 2012; Sporns, 2012, 2013a; Fornito et al., 2013; Smith et al., 2013a,b), it has not completely displaced classical signal processing methods. Even studies that focus on the frequency domain however concentrate almost exclusively on temporal frequency content of predefined regions (Chang and Glover, 2010; Kalcher et al., 2014; Yuan et al., 2014), voxel weightings (e.g., ICA components, Jafri et al., 2008; Erhardt et al., 2011b; Beckmann, 2012) or individual voxels (Van Someren et al., 2011; Ciuciu et al., 2012; Zalesky et al., 2012; Boubela et al., 2013; Boyacioglu et al., 2013) with efforts to consider spatial structure mostly in the realm of identifying voxel collections that have similar temporal frequency domain properties (Muller et al., 2007; Lohmann et al., 2010; Craddock et al., 2012; Thirion et al., 2014). Existing results from the temporal domain point to age and gender effects in lower temporal frequency bands (0.00–0.20 Hz. decreasing with age, 0.00–0.05 Hz. favoring males) (Allen et al., 2011), and to low-frequency (<0.10 Hz.) aberrations in schizophrenia patients (Hoptman et al., 2010; Calhoun et al., 2011; Turner et al., 2012; Yu et al., 2013). Some studies (Garrity et al., 2007; Calhoun et al., 2008) have demonstrated that functional network timecourses of schizophrenia patients have less low frequency temporal power (0.00–0.10 Hz.) and greater higher frequency temporal power (0.10–0.30 Hz.) than those of healthy controls. Our findings are consistent with evidence from the temporal domain, but elaborate these phenomena into the realm of scaled spatial patterning, giving a richer picture of how generic non-located spatiotemporal patterns of hemodynamic activity can differ across populations of interest. For example, our analysis indicates that while low-band temporal power decreases with age and is higher for males than females, this effect is concentrated in middle-band spatial frequencies for men while pervading nearly all spatial frequencies for age, suggesting greater impact of age than gender on the rapidity with which both broad diffuse activity and highly spatially variable local patterns develop and recur.

## Methods and materials

### Participants

Eyes-closed resting state fMRI data (5.38 min; *n* = 262) from six Siemens 3T scanners was collected on 127 SZ patients (mean age ± *SD* = 38.50 ± 11.83, 94 males) and 135 healthy volunteers (mean age ± *SD* = 37.54± 11.27, 96 males) matched as much as possible for age, sex, handedness, and race distributions, recruited from six sites, who participated in the study (Table 1). Inclusion criteria for the patients were an SZ diagnosis based on the Structured Clinical Interview for DSM-IV-TR Axis I Disorders (SCID-I/P) (First et al., 2002). All patients were clinically stable on antipsychotic medication for at least 2 months, and had an illness duration of minimally 1 year. Written informed consent was obtained from all study participants, and included permission to share de-identified data between the centers and with the wider research community.

**Table 1:** **Demographic Information**.

**Subject demographic information**			
**Schizophrenia patient (SZ)**	**127**	**Healthy control (HC)**	**135**
Male	190 (*SZ* = 94)	Female	72 (*SZ* = 33)
Ages 18–30	86 (*SZ* = 42)	Ages 31–60	176 (*SZ* = 85)

### Imaging parameters

Imaging data for the six sites used in this study was collected on a 3T Siemens Tim Trio System scanner. Resting state fMRI scans were acquired using a standard gradient-echo echo planar imaging paradigm: FOV of 220 × 220 mm (64 × 64 matrix), *TR* = 2 s, *TE* = 30 ms, *FA* = 770, 162 volumes, 32 sequential ascending axial slices of 4 mm thickness and 1 mm skip. Subjects had their eyes closed during the resting state scan.

### Data preprocessing

The three translation and three rotational head movement parameters for each subject were checked for maximal overall movement relative to the first image. Subjects who moved more than 4 mm. were excluded from the analysis. Further data-driven processing steps (Friedman et al., 2008; Turner et al., 2013) allowed removal of additional subjects whose scans were likely to contain significant motion contamination. The images were preprocessed using the MRN automated analysis pipeline (Bockholt et al., 2009), whose steps are conducted in SPM 5 (http://www.fil.ion.ucl.ac.uk/spm) as follows: Motion correction to the first image using INRIalign; slice timing corrected to the middle slice; and normalization to MNI space, including reslicing to 3 × 3 × 3 mm. voxels; despiking (as implemented in AFNI). Although a spatial smoothing step is typically applied to normalized despiked data, we do not implement this step as it imposes properties on the spatial frequency domain which in our case is a primary object of the present study.

### Spatiotemporal spectral profiles (STSPs)

We use Matlab's implementation of the n-dimensional fast Fourier transform to transform each subject's 63 × 53 × 46 × 162 masked, preprocessed data into the frequency domain and take squared magnitudes of the Fourier coefficients. The result is a 32 × 27 × 23 × 81 array. The raw coefficient magnitudes *f*_*i,j,k,l*_ are normalized with respect to the overall distribution of each subject's spatiotemporal power:

$${\tilde{f}}_{i,j,k,l}=\frac{\left\{\#\left(i^{\prime },j^{\prime },k^{\prime },l^{\prime }\right):f_{i,j,k,l}\leq f_{i^{\prime },j^{\prime },k^{\prime },l^{\prime }}\right\}}{32\times 27\times 23\times 81}$$

**Figure 2:**
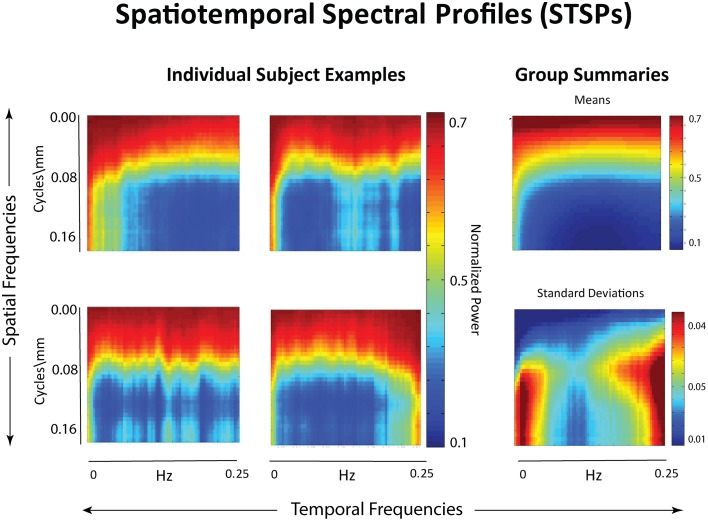
**Left:** Examples of individual spatiotemporal spectral profiles (STSPs); **Right:** Means for entire sample (top) and standard deviations (bottom).

**Figure 3:**
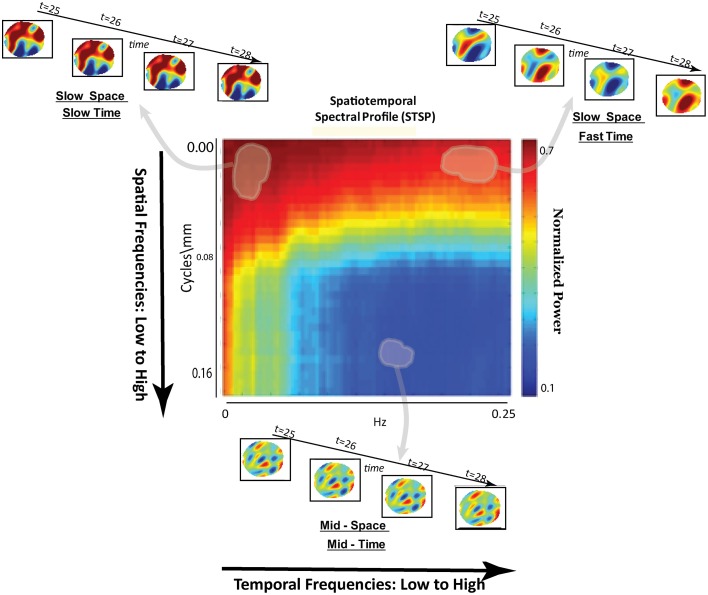
**Schematic breakdown of spatiotemporal spectral profile (STSP) with arrows to examples of subject data filtered for content in indicated spatiotemporal frequency bands**.

**Figure 4:**
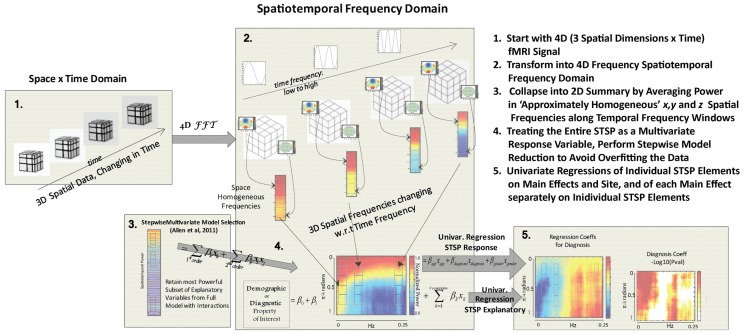
**Schematic outline of methods**.

**Figure 5:**
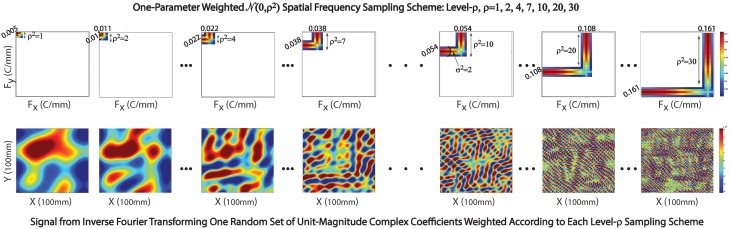
**Sampling scheme in exhibited in 2D setting. Top Row**: The Gaussian weighting scheme centered at (x,y) spatial frequencies with indices ρ = 1, 2, 4, 7, 10, 20, and 30; **Bottom Row**: Corresponding 2D spatial signals generated by inverse Fourier transforming a random set of unit-magnitude complex coefficients, weighting according to the sampling scheme displayed immediately above.

This particular normalization was chosen because we are primarily interested in the importance of certain frequency quadruples *relative to* the way power is distributed across a subject's entire 4D spectrum. The full 4-dimensional array of normalized magnitudes, does not provide a significant reduction of input data and is too high-dimensional to visualize. We therefore concentrate on spatial periods of roughly homogeneous extent in *x*, *y*, and *z*, essentially using a 1-parameter snapshot to convey the presence of spatial patterning at various scales. We attempt to capture this information in a 32 × 81 matrix Σ that we call the *spatiotemporal spectral profile* (STSP) of the subject's 4D scan (Figures 2–4). The (*r*, *t*)^*th*^ element of Σ is the average, in a radius-2 window about the *t*^*th*^ temporal frequency, of the normalized spatial power along the weighted radius-2 cubical hypertubes through $\tilde{f}$_*r*,1,1,*t*_, $\tilde{f}$_1,*r*,1,*t*_ and $\tilde{f}$_1,1,*r*,*t*_. These three coefficients, when equal in magnitude, represent the power in radially symmetric 3D sinusoids of spatial frequency *s*. The three radially symmetric coefficients at each spatial frequency are weighted maximally, at 1; weights on coefficients along the radius-2 cubical hypertube through each decay as a Gaussian of standard deviation *r* longtitudinally and as a Gaussian of standard deviation 2 transversally (Figure 5). The longtitudinal decay continues until the hypertubes intersect at the spatial frequency diagonal $\tilde{f}$_*r,r,r,t*_, beyond which the weights are set to be zero. In equation form, the (*r,t*)^*th*^ entry of the STSP is given by:

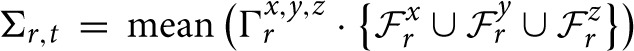



where Γ^*x,y,z*^_*r*_ is the 3D Gaussian weighting function centered at spatial frequency index *r* and the union of 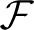
^*x*^_*r*_, 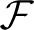
^*y*^_*r*_, and 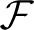
^*z*^_*r*_ contains all elements $\tilde{f}$_*i,j,k,t*_ of the normalized 4D spectrum (including those in the temporal frequency window *t* ± ϵ) on the 3D radius-ϵ “partial cross” through spatial frequency *r*. Specifically, the 3D Gaussian weighting function Γ^*x,y,z*^_*r*_(*i*, *j*, *k*, *t*) has the form:

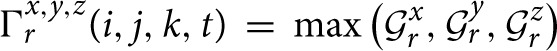



where

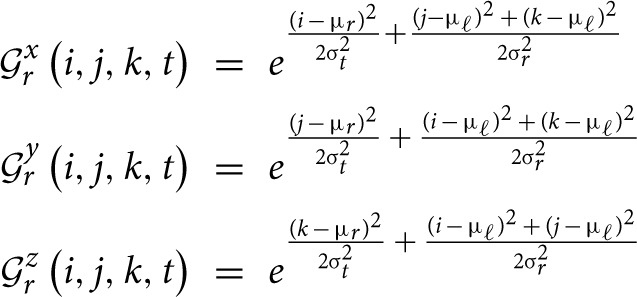



and μ_ℓ_ = 1, μ_*r*_ = *r*, σ_*t*_ = 2, σ_*r*_ = *r*. The directional weighting functions 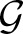
^*^_r_ (*i*, *j*, *k*, *t*) are products of two Gaussians, one that decays with distance from *r* in the superscripted dimension and one that decays with distance from the lowest spatial frequency index in the other two dimensions. 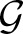
^*x*^_*r*_(*i*, *j*, *k*, *t*), for example, is the product of the standard deviation 2 Gaussian transverse to the plane *x* = *r*, and the 2D standard deviation *r* Gaussian that decays with distance from both (*y,1*) and (*1, z*) defined within any (*y,z*)-plane (including that determined by *x* = *r*). At each *r* and *t* (and for ϵ ∈ {0, 1, 2}) the weighting function Γ^*x,y,z*^_*r*_ is applied to the normalized 4D Fourier magnitudes $\tilde{f}$_*i,j,k,t*_ in each of 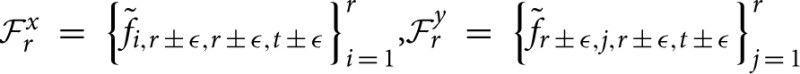 and 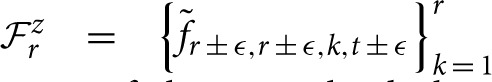, and Σ_*r,t*_ is the mean of the union of these weighted elements of the normalized 4D spectrum. Averages involving higher spatial frequencies are taken in neighborhoods of the fastest available frequency in each spatial dimension less than or equal to *r* (shrinking the cubical radius to accommodate edges as necessary). While the idea of this 1-parameter spatial frequency sampling scheme is to capture periodic patterns of roughly homogeneous extent in *x*, *y*, and *z*, it should be noted that the differing maximal span of gray matter in each direction distorts this objective somewhat: the *r*^*th*^ indexed spatial frequency in each Euclidean direction references a different frequency in terms of cycles/voxel or cycles/mm. For example, the 23^*rd*^ frequency has voxel-periodlength 2 in the *z*-direction and voxel-periodlength approximately 2.78 ≈ 2$\frac{32}{23}$ in the *x*-direction. Elements of the *r*^*th*^ row of Σ all summarize power in different temporal frequency bins of spatial frequency triples that are *simultaneously* approximately $\frac{1}{2}\frac{k}{32}$ cycles/voxel, or $\frac{1}{6}\frac{k}{32}$ cycles/mm (voxels are 3 mm^3^), in the *x*, *y*, and *z* directions.

### Spatial spectral profiles (SSPs)

Although STSPs are the main focus of our analysis, we employ a purely spatial version of this construction to characterize functional network 3D spatial maps (SMs), obtained from a group independent component analysis (GICA) applied to our data (Damaraju et al., 2012). The spatial spectral profile (SSP) of the 63 × 53 × 46 network SMs is the length 32 = $\left\lceil \frac{63}{2}\right\rceil$ vector whose *r*^*th*^ element is the average value in the weighted radius-2 hypertubes through the (*r*, 1, 1), (1, *r*, 1) and (1, 1, *r*)^*th*^ element of the normalized spectrum $\tilde{F}=\;{\left\{{\tilde{f}}_{i,j,k}\right\}}_{i,j,k\;=\;1}^{32}$ of the 3D network maps. The procedure is exactly as described in Section Spatiotemporal Spectral Profiles (STSPs) above, except there is not an additional time dimension.

### Model selection and univariate tests

Starting with a full model consisting of 49 explanatory variables: age, gender, diagnosis, mean frame displacement (motion), site indicators for each of the six sites and all pairwise interactions we implement multivariate backward model selection utilizing thethe Matlab-based MANCOVAN toolbox (http://mialab.mrn.org/software/mancovan/) (Allen et al., 2011). The site indicators were treated as a group: if one survived model selection then all would be retained. This procedure yielded a reduced model involving only first-order main effects (age, gender, diagnosis) plus the six site indicators. Having selected a parsimonious model based on overall multivariate explanatory power, we then estimate parameters separately for each of the spatiotemporal frequency combinations σ_*r,t*_ ∈ Σ. So for each (*r*, *t*) ∈ {1, 2, …, 32} × {1, 2, …, 81} we estimate 2692 = 32 × 81 univariate models:

$$\sigma_{r,t}=\beta_{0}^{\left(r,t\right)}+\sum_{k\in \left\{\text{age},\text{ gender},\text{ diagnosis}\right\}}\beta_{k}^{\left(r,t\right)}X_{k}+\sum_{\text{site}\in \left\{1,2,..,6\right\}}\beta_{\text{site}}^{\left(r,t\right)}X_{\text{site}}$$

### Spatially-filtered FNCs

The fMRI dataset utilized in this study recently underwent a thorough group ICA-based functional network connectivity (FNC) analysis (Damaraju et al., 2012), yielding 47 meaningful functional network SMs and associated timecourses (TCs). One way to incorporate spatial frequency domain information into an FNC framework is to evaluate correlative properties of network timecourses on SMs that have been filtered for frequency content in restricted spatial frequency bands. We call this a spatially-filtered FNC (SFFNC) analysis. There is a body of work (Cordes et al., 2001a,b; Sasai et al., 2014; Tenney et al., 2014) in which correlations are measured on timecourses whose temporal frequency content is band-filtered. SFFNCs are the spatial frequency domain analog, in which network SMs are spatially band-filtered, yielding a distinct collection of network timecourses associated with each spatial frequency band. The SFFNCs used here are the result of filtering all 100 network SMs (the 47 functional networks and the remaining 53 determined to be artifactual) obtained from the GICA reported in Damaraju et al. (2012) for their content in each of the following overlapping spatial frequency bands:

**Table:** 

**Band #**	**Cycles/mm**
1	[0.0000, 0.0239]
2	[0.0119, 0.0358]
3	[0.0239, 0.0477]
4	[0.0358, 0.0716]
5	[0.0477, 0.0835]
6	[0.0596, 0.0835]
7	[0.0716, 0.0954]
8	[0.0835, 0.1074]
9	[0.0964, 0.1193]
10	[0.1074, 0.1312]
11	[0.1193, 0.1431]
12	[0.1312, 0.1551]
13	[0.1431, 0.1670]

To retain the important spatial structure that makes network SMs identifiable as objects with functional roles, the one-parameter spatial filtering applied here is more inclusive than the analogous process used to produce STSPs from subject spatial data. The filter for spatial frequency band [*r*_1_, *r*_2_] cycles/mm consists of unweighted frequencies with either *x*-direction restricted to [*r*_1_, *r*_2_] or *y*-direction restricted to [*r*_1_, *r*_2_]or *z*-direction restricted to [*r*_1_, *r*_2_]. Spatially-filtered network timecourses (SFTCs) for each spatial frequency band are produced by regressing subject data on the 100 filtered SMs The SFFNC at each spatial frequency band is the correlation matrix for the 47 functional network SFTCs produced by regression on SMs filtered for content in that band.

## Results

Our approach (Figure 4) differs meaningfully from the cross-network correlational analysis typically applied to fMRI data (Jafri et al., 2008; Rubinov and Sporns, 2010; Erhardt et al., 2011b; Kaiser, 2011; Power et al., 2011; Beckmann, 2012; Smith et al., 2013a). Its starting point is a 2D spatiotemporal frequency profile Σ of the full 4D image volume, *in toto* (Figures 2, 3). This profile summarizes the characteristic temporal rates at which different scales of generic, non-located spatial activation fluctuate and recur through the 3D brain volume. It is quantifying the relative contributions of 3D spatial intensity patterns of roughly homogeneous directional periodlengths moving at different temporal frequencies through the 4D fMRI signal. The coefficients from a 4D Fourier decomposition inherently capture spatial and temporal frequencies *simultaneously*: the magnitude of the (*i*, *j*, *k*, *t*)^*th*^ coefficient is the amount of power in the 4D signal living jointly in the *i*^*th*^ *x*-direction spatial frequency and the *j*^*th*^ *y*-direction spatial frequency and the *k*^*th*^ *z*-direction spatial frequency and the *t*^*th*^ temporal frequency (refer to Methods Section for details).

### Age, gender, and schizophrenia diagnosis as predictors of spatiotemporal spectrum

**Figure 6:**
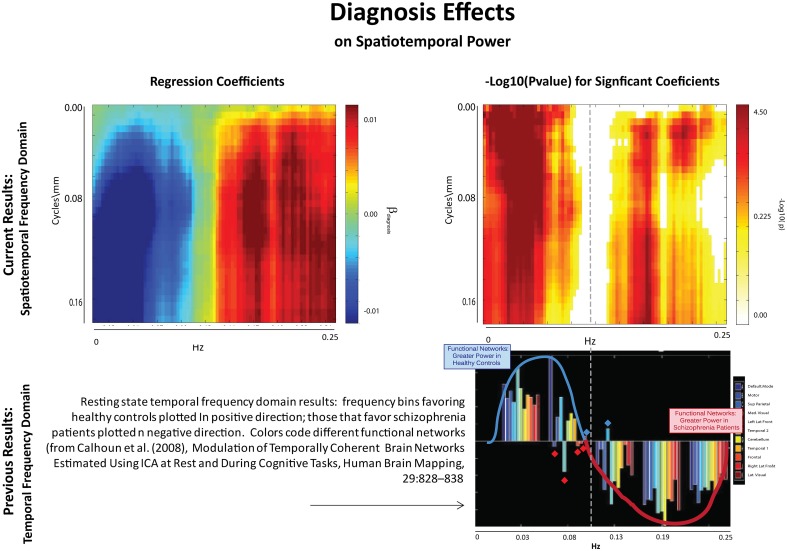
**Results of Univariate Regression of STSP elements on Main Effects. Top Left**: Coefficients (red shades are SZ > HC; blue shades are HC > SZ) for diagnosis, where schizophrenia patients are coded as ones and healthy controls as zeros; **Top Right**: –log_10_(p) of univariate *p*-values that survive FDR correction at the α = 0.05 significance level. **Bottom**: Figure displaying prior results from the temporal frequency domain. Significant differences between healthy controls (positive direction) and schizophrenia patients (negative direction) in given temporal frequency bin for various functional networks (from Calhoun et al., 2008).

**Figure 7:**
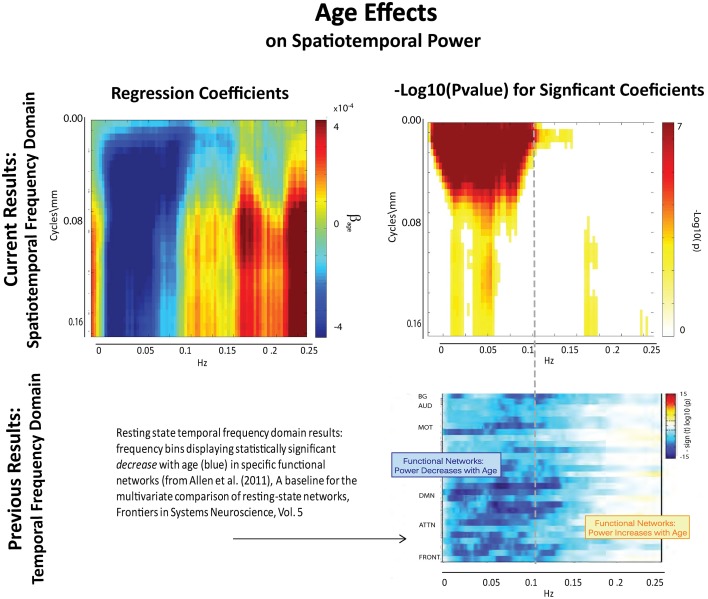
**Results of Univariate Regression of STSP elements on Main Effects. Top Left**: Coefficients (red shades indicate positive correlation with age; blue shades indicate negative correlation with age) for age, **Top Right**: −log_10_(p) of univariate *p*-values that survive FDR correction at the α = 0.05 significance level. **Bottom**: Figure shows −sign(t)log_10_(p) for temporal frequency bins with power declining statistically significantly with age in functional networks indexed along the y-direction and clustered according to their functional role (from Allen et al., 2011).

**Figure 8:**
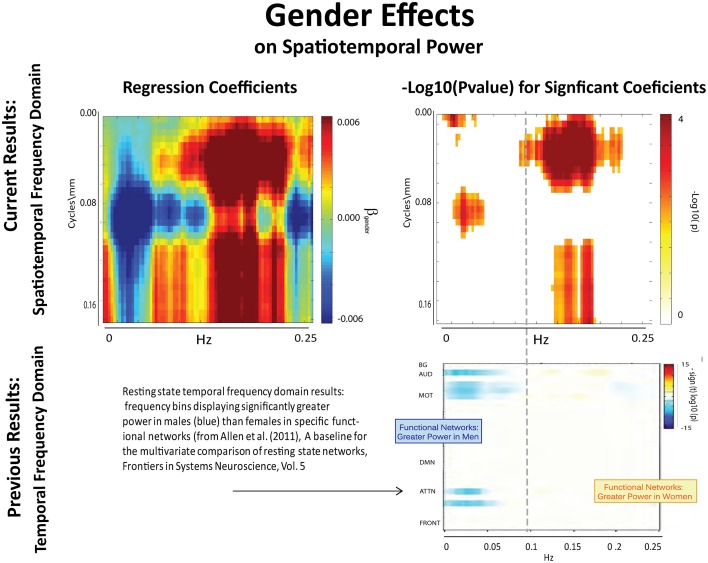
**Results of Univariate Regression of STSP elements on Main Effects. Top Left**: Coefficients (red shades are F > M; blue shades are M > F) for gender, **Top Right**: −log_10_(p) of univariate *p*-values that survive FDR correction at the α = 0.05 significance level. **Bottom**: Figure shows −sign(t)log_10_(p) for temporal frequency bins with greater power in males than females in functional networks indexed along the y-direction, and clustered according to their functional role (from Allen et al., 2011).

The results of this analysis (Figures 6–8) indicate striking differences in spatiotemporal organization of brain activity that correlate with presence of diagnosed schizophrenia, as well as with gender and age. Comparing schizophrenia patients with controls, we see a sharp distinction across almost all spatial frequencies developing at approximately 0.10 Hz (Figure 6: top left, top right). This finding is consistent in spirit with growing evidence of greater power in higher temporal frequencies for patients compared with controls at the voxel and network levels (Garrity et al., 2007; Calhoun et al., 2008, 2011; Skudlarski et al., 2010). It should be remembered that the temporal frequencies reported here represent rates of change and recurrence of *spatial patterns* of various scales. Generic dynamics of this type obviously underlie network-level behavior, but the time-identified dimension of a signal's four dimensional spatiotemporal spectrum cannot be directly translated into knowledge of one dimensional temporal spectra at either the indexed voxel or network levels. The stark dividing line at temporal frequencies near 0.10 Hz., spanning most spatial frequencies, observed for diagnostic status also characterizes the contribution of increasing age to spatiotemporal spectral power (Figure 7). The pattern for gender is more complicated: males dominate mid-range spatial frequencies over a wide range of temporal frequencies; females have more power in high spatial frequencies through most temporal frequencies, and more power in all but the mid-range spatial frequencies at temporal frequencies greater than 0.10 Hz (Figure 8).

### Integration with network and correlation-based approaches

The present work captures fundamentally different information about brain activation than is typically analyzed in fMRI studies (van den Heuvel and Pol, 2010; Power et al., 2011; Tomasi and Volkow, 2012; Smith et al., 2013b; Sporns, 2013b). Our analysis deals with properties of the *ambient* spatiotemporal activation environment within which networks and regions of interest perform their understood functional roles. Since the data treated here recently underwent a thorough FNC analysis (Damaraju et al., 2012), we happen to have a set of meaningful functional network SMs and associated timecourses (TCs) with which to probe the indirect connections between the two approaches.

**Figure 9:**
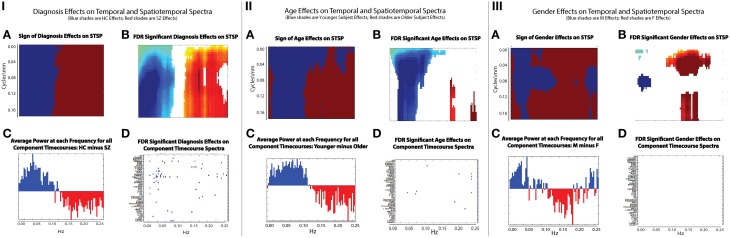
**(IA)** Sign of diagnosis effects Figure 6 (top left); **(IB)** Diagnosis effects on STSP that are significant at the α = 0.05 level after FDR correction; **(IC)** Average power of all 47 functional network timecourses from Allen et al. (2011): healthy controls minus schizophrenia patients; **(ID)** Diagnosis effects on functional network timecouse spectra that are significant at the α = 0.05 level after FDR correction; **(IIA)** Sign of age effects Figure 8 (top left); **(IIB)** Age effects on STSP that are significant at the α = 0.05 level after FDR correction; **(IIC)** Average power of all 47 functional network timecourses from Allen et al. (2011): younger subjects (18–34 year olds) minus older subjects (35–60 year olds); **(IID)** Age effects on functional network timecouse spectra that are significant at the α = 0.05 level after FDR correction; **(IIIA)** Sign of gender effects Figure 7 (top left); **(IIIB)** Gender effects on STSP that are significant at the α = 0.05 level after FDR correction; **(IIIC)** Average power of all 47 functional network timecourses from Allen et al. (2011): males minus females; **(IIID)** Gender effects on functional network timecouse spectra that are significant at the α = 0.05 level after FDR correction.

There are two dimensions along which it is tempting to make comparisons. Since our work takes place in the frequency domain, we first consider the network timecourse spectra (Figure 9). The effect of diagnosis on STSP breaks more cleanly along the temporal frequency axis than either age or gender (Figure 9(I.A)) shows just the sign of the effect, i.e., whether the effect favors healthy controls (blue) or schizophrenia patients (maroon), regardless of statistical significance and group differences in average power taken over all networks at different temporal frequencies (Figure 9(I.C)) show nearly perfect alignment with the effect of diagnosis on STSP in the temporal frequency dimension. Even along this temporal frequency dimension however, the effect of diagnosis on STSP displays much greater statistical significance than (Figure 9(I.B)) than does the diagnosis effect on individual network timecourse spectra (Figure 9(I.D)) indicating that patterns of temporal recurrence localized to specific spatial frequency bands is a form of information to which certain explanatory variables (all of those included in this study) “tune” their effects.

**Figure 10:**
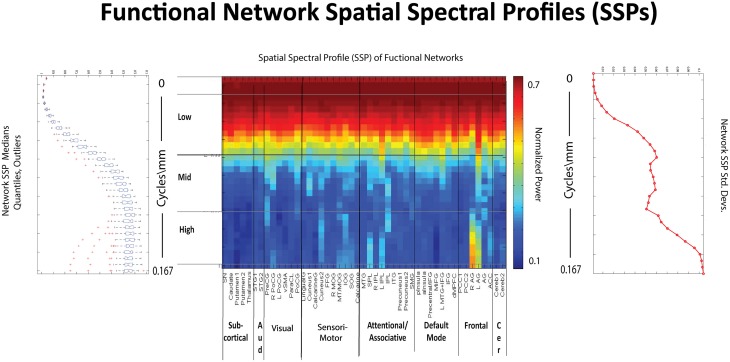
**Spatial Spectral Profiles (SSPs) of the 47 functional networks identified in our sample using Group Independent Component Analysis (GICA) (Allen et al., 2011). Left:** Spatial power distribution: medians, quantiles, and outliers for all 47 networks. **Middle**: SSPs of each of the 47 networks in labeled columns. Functional clusters indicated along bottom horizontal. Rough spatial frequency bands indicated along the left vertical axis. **Right**: Standard deviations of SSP spatial power over all 47 networks.

The directional effect of age (blue is negative; maroon is positive) on STSP (Figure 9(II.A)) actually extends into higher temporal frequencies than is evident in the group average power difference taken over functional networks at different temporal frequencies (Figure 9(II.C)). The extended directional effect in temporal frequency is restricted to lower spatial frequency bands in which the network SMs carry most of their power (Figure 10). The inability of network timecourse spectra to reflect temporal activation localized within specific spatial frequency bands seems both to dilute the power of the age effect (Figures 9(II.B,D)) and to obscure the range of the directional effect in the temporal frequency domain.

The directional effect of gender on STSP (Figure 9(III.A)) patterns heavily along the spatial frequency axis, making comparison with results drawn exclusively from the temporal frequency domain (Figure 9(III.C)) less straightforward. The picture with gender and the spatial frequency domain is complex, and will be explored more thoroughly in forthcoming work. We see, for example, that the effects favoring males (blue) are evident in the network timecourse spectra even when they occur in restricted spatial frequency bands. However, effects on STSPs that favor females (maroon) are reflected in the temporal frequency domain primarily when they are nearly uniform over spatial frequencies. As was true with age and diagnosis, we also observe that gender effects on patterns of temporal recurrence localized to specific spatial frequency bands (Figure 9(III.B)) are much more statistically powerful than gender effects on network timecourse spectra (Figure 9(III.D)).

**Figure 11:**
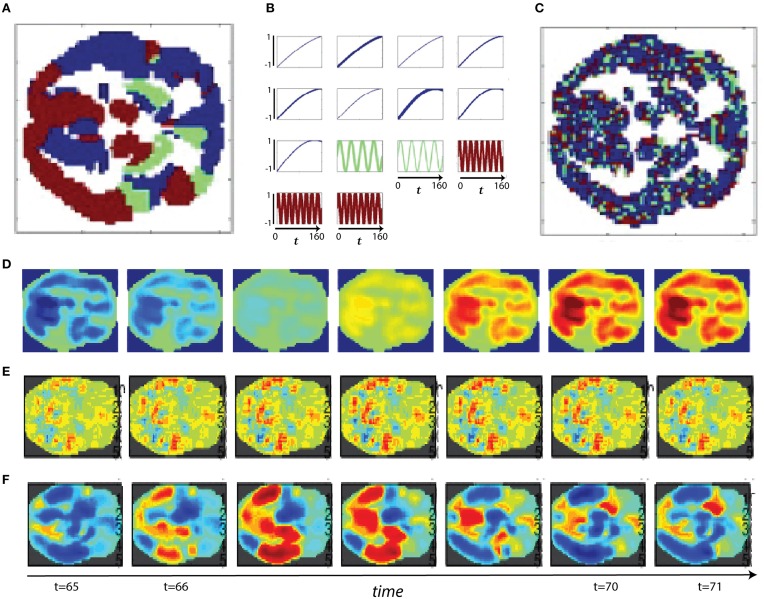
**(A)** Slower spatial frequency slice parcellation; **(B)** Slower temporal frequency signals randomly assigned to regions of **(A)**, line thickness scales with region size; **(C)** Faster spatial frequency slice parcellation; **(D)** Example of slower spatial frequency activation pattern based only on differential timecourse amplitudes; **(E)** Example of faster spatial frequency activation pattern based only on variation in the timecourse phases; **(F)** Example of slower spatial frequency activation pattern based only on differing timecourse frequencies.

**Figure 12:**
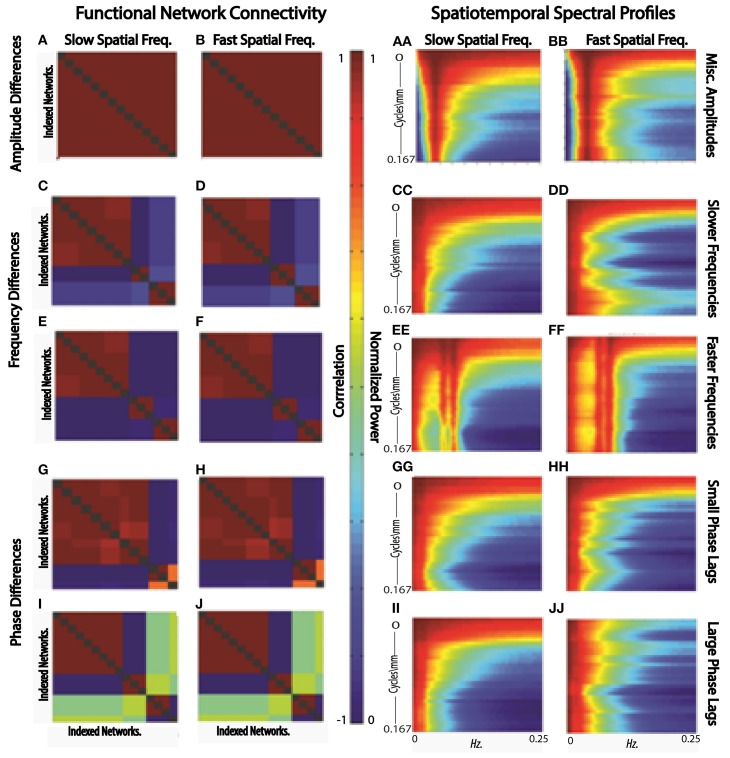
**FNCs (A,B) and STSPs (AA,BB) of simulated slow (A,AA) and fast (B,BB) spatial frequency data in which region timecourses vary only in** ***amplitude***; **FNCs (C,D,E,F) and STSPs (CC,DD,EE,FF) of simulated slow (C,E,CC,EE) and fast (D,F,DD,FF) spatial frequency data in which region timecourses vary only in** ***frequency*****[slow frequency regime (C,D,CC,DD)]; mixed frequency regime (E,F,EE,FF); FNCs (G,H,I,J) and STSPs (GG,HH,II,JJ) of simulated slow (G,I,GG,II) and fast (H,J,HH,JJ) spatial frequency data in which region timecourses vary only in** ***phase*** **(small phase lag regime G,H,GG,HH; large phase lag regime I,J,II,JJ)**. Parcellations and simulated data is identical for each letter/double-letter combination. Otherwise, the parcellations and signals have properties indicated, but represent a distinct runs of the simulation.

Comparisons between the spatiotemporal spectral analysis (STSPs) presented here and those based correlative network timecourse behavior (FNCs) can be accomplished only indirectly. The temporal phase information that underpins correlative FNC analysis is not explicitly accounted for in STSPs. Moreover, network timecourses contain neither explicit information about distributive patterns in space nor about phase-blind matching of signal frequency or amplitude (Figures 11, 12). To investigate implicit connections between STSPs and FNCs, we employ the SFFNCs detailed in Methods. Continuing with the predictive model used for the STSP analysis, we regress each network-pair correlation in an SFFNC on age, gender, diagnosis. The results do not have a straightforward interpretation, but do indicate that the cross-network correlative properties of functional network timecourses are strongly affected by the spatial frequency content of estimated brain networks.

**Figure 13:**
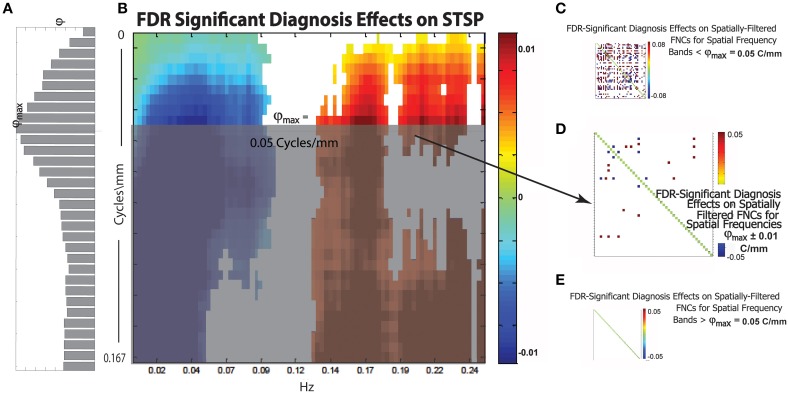
**(A)** Bar plot of φ(*r*), a weighted sum of the magnitudes of FDR-surviving diagnosis effects at spatial frequency *r* Cycles/mm (the maximum φ_*max*_ of this function occurs at approximately $\hat{r}$ = **0.05** Cycles/mm); **(B)** Diagnosis effects on STSP that are significant at the α = 0.05 level after FDR correction; **(C)** Representative FDR-significant diagnosis effects on spatially-filtered FNC for spatial frequency bands [r_1_, *r*_2_] with r_1_ < *r*_2_ ≤ $\hat{r}$; **(D)** FDR-significant diagnosis effects on spatially-filtered FNC for spatial frequency band $\hat{r}$ ± 0.01 Cycles/mm; **(E)** Representative FDR-significant diagnosis effects for spatial frequency bands [r_1_, r_2_], $\hat{r}$ < *r*_1_ < *r*_2_; network order along axes of displayed SFFNCs is identical to that in prior and subsequent figures.

Diagnosis was the explanatory variable whose effect on STSPs was most temporally determined (Figure 9(I.A)). In such a case it would seem that space is nearly irrelevant, at least with respect to effect directionality. We see however that subtle differences in effect strength across spatial frequencies can be reflected in SFFNCs (Figure 13), even in the case of an explanatory variable such as diagnosis whose directionality is nearly uniform in space. Further exploration of the relationship between SFFNCs and spatial frequency content of functional networks can be found in the Ancillary Results subsection below.

### Ancillary result: simulations illustrating strengths and limitations of FNC and STSP analyses

Connectivity-based methods and spatiotemporal spectral techniques such as the one we introduce here are sensitive to different features of fMRI data. We present a set of stylized (i.e., highly simplified, non-biologically realistic) simulations to clarify and illustrate certain features of spatiotemporal signals that are detected or overlooked by FNC and STSP analyses. The temporal behavior of networks can differ in signal amplitude, frequency or phase. Moreover, the voxel intensities that define networks can distribute differentially in space, presenting in sparse spider-like patterns or broad regions of homogeneous intensities. For each example we start with two parcellations of a medial axial slice into distributed networks. One such parcellation is dominated by lower spatial frequencies (Figure 11A), the other by higher spatial frequencies (Figure 11C). Every voxel of a given network is assumed to behave identically–a simplification that is intended to clarify methodological strengths and weaknesses, not to mimic biological realities. Signals varying along some parameter of interest, say amplitude (Figure 11B), are then assigned randomly to a set of networks in the parcellation.

In the first scenario we investigate, a brain^1^ is assumed to consist of distributed networks whose activation, and degree of co-activation, are expressed entirely in signal amplitude. The whole brain operates at a common set of temporal frequencies/phases. Networks here activate and co-activate based only on their relative timecourse amplitudes. FNC provides no information about which networks are co-activating in this simplified situation, nor about how the co-activating networks occupy space (Figures 12A,B). STSPs show that networks are co-activating (with respect to signal amplitude) at temporal frequencies around 0.01 Hz (Figures 12AA, BB), and also indicate that in one case (Figure 12AA) the co-activating networks are, spatially, dominated by lower spatial frequencies, while in the other (Figure 12BB), the co-activating networks exhibit noticeably less low spatial frequency power, and more high spatial frequency power in the relevant temporal frequency interval.

In our second scenario, the “brain” consists of distributed networks whose activation, and degree of co-activation, are expressed entirely through temporal signal frequency. The whole brain operates at a common set of amplitudes and phases. Networks here co-activate based only on similarity of timecourse frequency. FNC provides no information about how the co-activating networks occupy space in this situation (Figures 12C–F) and no real sense that in one sub-case (Figures 12C,D) the frequencies are very slow, indicating a kind of *dormant coactivation* while in the other sub-case (Figures 12E,F) co-activating networks are authentically active, producing signals with some frequency content in the 0.1 Hz range. STSPs clearly show that co-activating networks in one case (Figures 12CC,EE) are characterized by lower spatial frequencies, and in the other case (Figures 12DD,FF) by higher spatial frequencies. Moreover, the STSPs clearly separate the dormant (very low temporal frequency) and active (higher temporal frequency) coactivation sub-cases (Figures 12CC,DD vs. Figures 12EE,FF).

The “brain” of the last scenario consists of distributed networks whose activation and degree of co-activation, are expressed entirely through temporal phase. The whole brain operates at a common set of amplitudes and frequencies. Networks here co-activate based only on similarity of timecourse phase. As we might expect, since correlation is a rough measure of phase-locking, FNC is fairly informative here. It does not, of course, give much information about how co-activating networks occupy space information about how the co-activating networks occupy space (Figures 12G,I vs. Figures 12H,J), but the sub-case in which relative phase lags are small shows many more networks co-activating than does the sub-case in which larger phase lags are included (Figures 12G,H vs. Figures 12I,J). For the small relative phase-lag sub-case, STSPs are unable to detect much about co-activation patterns or spatial frequency properties of the co-activating networks (Figures 12GG,HH). Larger temporal phase lags enable STSPs to more clearly pick up spatial frequency differences networks (Figures 12II,JJ).

### Ancillary results: spatially filtered FNCs and network spatial spectra

The relationship between spatiotemporal frequency domain properties and nodal network timecourse correlations (FNCs) is complex. More than one set of properties can obtain in a collection of network SMs and timecourses associated to data that produces a given STSP. Similarly, the timecourse correlation structure for a given set of network SMs does not strongly constrain STSP. Most obviously, the correlation between two timecourses says nothing about their frequency decompositions. SFFNCs are hybrid objects that straddle the two approaches, offering opportunities for insight into how they interact.

#### SFFNC diagnosis effects as a function of spatial frequency band of the filter

We introduce the function φ:(0, 1) → ℝ^+^ (Figure 13A), which is intended to capture the relative importance of spatial frequencies around $\frac{\text{r}}{6}$ Cycles/mm in the overall 4D signal diagnosis effect. For *r* ∈ (0, 1), φ(*r*) is the sum of the magnitudes of the FDR-surviving STSP diagnosis effects (Figure 13B) at $\frac{\text{r}}{6}$ Cycles/mm rescaled by the average proportion of functional network SSP spatial power at that spatial frequency. The idea here is to give more “credit” for diagnosis effects in spatial frequency bands that contribute heavily to the SMs we are filtering in order to perform the SFFNC analysis. The maximum, φ_*max*_, of φ should occur at a spatial frequency $\hat{r}$ exhibiting both strong STSP diagnosis effects and significant contribution to network SMs. Thus, we might expect SFFNC diagnosis effects to bear some relationship to values assumed by φ. This is in fact what happens. The diagnosis effects on SFFNCs taken on spatial frequency bands [*r*_1_, *r*_2_], *r*_1_ < *r*_2_ ≤ $\hat{r}$ (Figure 13C) are pervasive. We display one example but the others are visually indistinguishable from this case. There is a bifurcation at $\hat{r}$ = 0.05 Cycles/mm where φ_*max*_ is achieved. The SFFNC diagnosis effects on the first spatial frequency band including frequencies greater than $\hat{r}$ (Figure 13D) differs starkly from the SFFNCs both for lower spatial frequencies. (Figure 13C) and for higher spatial frequencies (Figure 13E). This indicates an important role for spatial frequency content even for explanatory variables whose directional effects are temporally determined.

#### SFFNC diagnosis effects and spatial frequency content of functional network spatial maps

**Figure 14:**
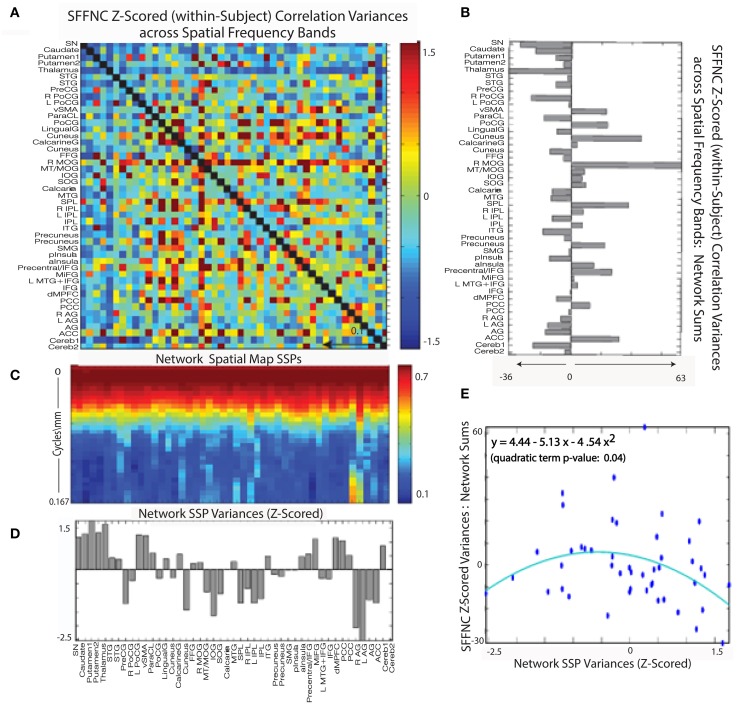
**(A)** Mean z-scored (within-subject) variances over spatial frequency bands of cross-network SFTC correlations; **(B)** Network-wise sums of the SFFNC variances from **(A)**; **(C)** Network spatial map SSPs; **(D)** Z-scored network SSP variances; **(E)** Scatterplot of summed z-scored network cross-frequency SFFNC variances against z-scored network spatial map SSP variances, indicating a roughly parabolic (y = 4.44 − 5.14x− 4.54x^2^, quadratic term *p* = 0.04 relationship between the two quantities.

The variance of network-pair SFTC correlations (Figure 14A) in SFFNCs evaluated over sliding windows through the spatial frequency domain (see Methods Section, subsection concerning Spatially Filtered FNCs) is a measure of the sensitivity of FNC correlation data to scaled properties of network spatial structure. We might expect that the variance of the SSP (Figure 14D) of a network would have some bearing on the sensitivity of the network's timecourses to spatial filtering of the network SMs (Figure 14B). This is not completely straightforward because each network's SFTC in a given spatial frequency band depends on how all the other spatial networks are affected by the spatial filtering. The sensitivity of a network's SFTC correlations to spatial map filtering should depend on a combination of the spectral variability of its own spatial map and the degree to which its spectral properties are similar to those of other networks. This latter aspect affects the likelihood that a network's role relative to other networks changes under spatial filtering, which in turn impacts the likelihood that the network SFTCs will correlate differentially with other network SFTCs according to the frequency band of the spatial filter applied to network SMs.

Figure 14G shows the a significant (convex) parabolic relationship distinctness of network SSP (as measured by the average distance of a given SSP from all the others) and SSP variance, i.e. network SMs with the highest and lowest spectral variability are most spectrally distinct from other networks in the L^1^ sense and those with average spectral variability are least distinct.

A combined role for SSP distinctness and variability in predicting SFFNC variances is supported by the significant (concave) parabolic relationship between network SFFNC variance with respect to spatial frequency band and full-spectrum network SSP variance (Figure 14E). This indicates that the highest and lowest variance SSPs, which are also the most distinct, belong to networks with relatively low SFFNC sensitivity to spatial frequency band, while those SSPs with more average variance, which also happen to be less distinct, belong to networks with high SFFNC sensitivity to spatial frequency band.

## Discussion

Our results show that broad organizing principles influencing the rate at which scaled spatial activation patterns recur in time differ across populations of interest, suggesting more complex interplay than is typically investigated between nodal network timecourses and the ambient multi-scaled hemodynamic activity from which they are distilled (Breakspear et al., 2006). We trade off resolution on correlation and nodal identity for an “aerial view” of scaled spatial activation patterns throughout the brain developing in time. While (temporal) phase synchrony, i.e., timecourse correlation, is not captured explicitly in this analysis, phase-locking between distributed spatial regions of various scales will appear as power at relevant spatiotemporal frequencies–where the set of relevant frequencies is modulated by phases and frequencies of the signals from complementary regions.

Correlational behavior *per se* is less directly identifiable in 4D spectral data. The STSPs we report here however does succeed in highlighting important features of brain activation that are obscured in conventional FNC analyses. For example (Figures 11, 12), the presence or absence of broad spatial frequency bands is detectable in this setting for spatial activation patterns resulting from variations in signal amplitude, frequency or phase. Standard FNC approaches cannot distinguish between the temporally correlated behavior of near-dormant regions and that of highly active regions. Different rates of temporal activation are very clearly exposed however in STSPs. Furthermore, while temporal phase differences are certainly captured explicitly in FNC analyses, the ways that phase-synchronized regions distribute in space (possibly differentially across populations) cannot be extracted from nodal timecourse correlations.

We find that previously reported temporal frequency domain results from schizophrenia patients (with respect to nodal network timecourses) (Garrity et al., 2007; Calhoun et al., 2008, 2011; Skudlarski et al., 2010) persist under the addition of a spatial dimension and actually pervade all scales of spatial organization, not merely those in which functional networks carry significant spatial power.

**Figure 15:**
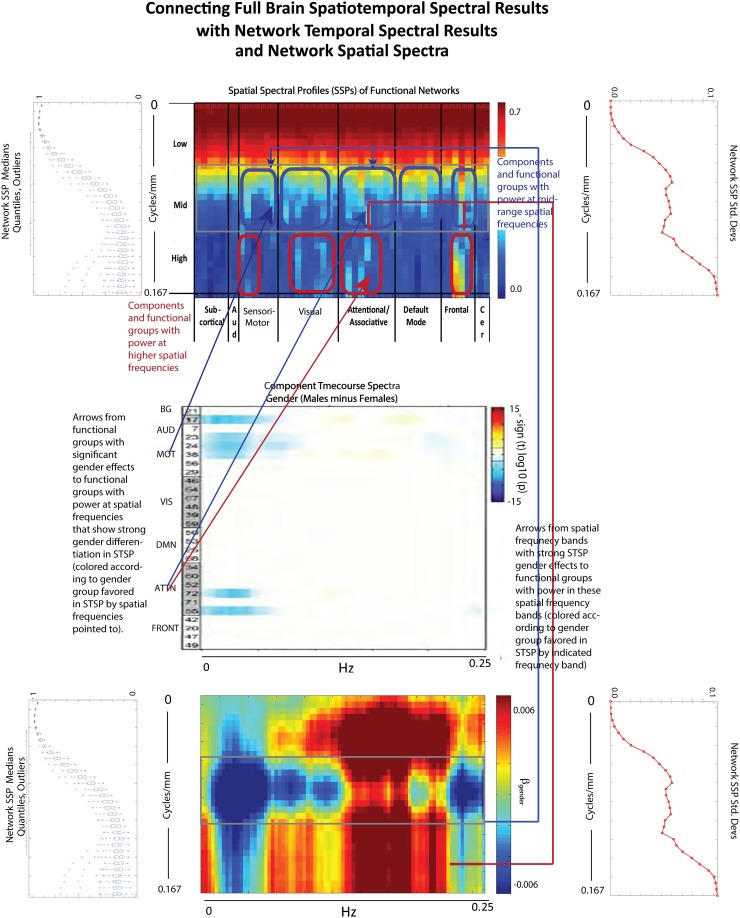
**Spatial Spectral Profiles (SSPs) of the 47 functional networks identified in our sample using Group Independent Component Analysis (GICA) (Allen et al., 2011). (Top Left, Bottom Left):** Spatial power distribution: medians, quantiles, and outliers for all 47 networks **(Top Middle)**: SSPs of each of the 47 networks in labeled columns. Functional clusters indicated along bottom horizontal. Rough spatial frequency bands indicated along the left vertical axis. **(Top Right, Bottom Right)**: Standard deviations of SSP spatial power over all 47 networks. **(Middle)**: Figure shows –sign(t)log_10_(p) for temporal frequency bins with greater power in males than females in functional networks indexed along the y-direction, and clustered according to their functional role (Damaraju et al., 2012) **(Bottom Middle)**: Results of univariate Regression of STSP elements on main effects.

The effect of schizophrenia diagnosis on STSP breaks cleanly along the temporal frequency axis, but this need not be the case. For example, the two genders affect STSP in frequency bands defined along different axes (see Figures 8, 15). Here we see higher temporal frequencies dominated by women while it is a middle band of spatial frequencies that is most associated with males. There are only a small number of networks and temporal frequencies for which male subjects showed significantly more network timecourse power than females (Allen et al., 2011). The networks involved are auditory, sensorimotor, and attentional, which–like all identified functional networks–have significant power in lower spatial frequencies, but also have non-negligible power in many mid-range and several high spatial frequencies. The temporal behavior of the components with non-negligible mid-range spatial power shows up in STSPs as favoring males at temporal frequencies including, but not limited to, those under 0.075 Hz. where males had more power in the network timecourse spectral analysis (Allen et al., 2011) (Figure 15). There are spatiotemporal frequency combinations that significantly favor females in the STSP regression analysis, although the network timecourse spectra have no frequencies in which females carry significantly more power. Moreover, the temporal dimension of the STSP alone does not separate males from females; there are spatial frequencies in which men have more power than women, and vice versa at most temporal frequencies in [0.00, 0.13] ∪ [0.22, 0.25] Hz.

The connections between STSPs of full-volume fMRI data and network connectivity analysis are indirect but intriguing. There are echoes in each analysis of results obtained by the other. In the case of an explanatory variable such as diagnosis, whose effects on STSPs are nearly uniform (directionally) over all spatial frequencies, the 0.10 Hz. temporal-frequency breakpoint in effect directionality is respected by network timecourse spectra (Figures 9(I.A,D)). Even in this rather pure example of temporal domain dominance of effect directionality, some additional observations apply:

1. It is only a small subset of networks and temporal frequencies for which the diagnosis effect on network timecourse spectra is statistically significant (Figure 9(I.D)), whereas the diagnosis effect is pervasive and significant across spatial frequencies and through most temporal frequencies on either side of the 0.10 Hz. breakpoint (Figure 9(I.B)).
2. The overwhelming significance and stark temporal frequency domain demarcation of diagnosis effect directionality obscures some variation over spatial frequencies in both effect magnitude and reach through the temporal frequency domain. This variation is indirectly evident, however, in the number of significant effects on SFFNCs performed in different spatial frequency bands (Figures 14C–E).

The present approach is one of many possible routes into more direct reckoning with structured (Euclidean) spatial patterns of brain activation evolving in time. It is our initial foray into this area, and our focus here was on transparency at each stage: the decomposition is into canonical familiar waveforms, leading to a 2D reduction whose heuristic underpinnings are relatively easy to visualize and interpret. There are many limitations to the current approach. It assumes for example that the fMRI signal is temporally and spatially stationary, a simplifying assumption that shows no evidence of being true in the case of resting state data (Hutchison et al., 2013; Calhoun et al., 2014; Leonardi et al., 2014; Tagliazucchi et al., 2014; Zalesky et al., 2014). Very simple progress on this front has already been reported (Calhoun et al., 2014; Miller and Calhoun, 2014) with a time-windowed version of the analysis presented here. There is also the matter of physiological confounds (heart-rate, respiration) whose role in higher temporal frequencies is actively debated. A spectral domain study such as ours allows one to focus on whichever frequency bands are of interest; results pertaining to lower temporal frequencies are not contaminated by the reported results from higher temporal frequencies. Also, although averaging effects in a study this size can overcome subject level spectral leakage, adding a windowing step (as in Welch's method) to our pipeline would further mitigate the potential for subject-level estimator bias to impact group results. Investigations of spatiotemporal scaling properties in fMRI data are one way of working directly with both spatial and temporal features of the data (Ribeiro et al., 2010; Expert et al., 2011; Ciuciu et al., 2012; Tagliazucchi et al., 2012). The 4D wavelet transform is one particularly promising vehicle for extending the current frequency domain approach toward a more comprehensive characterization of the 4D signal, one that is free of both spatial and temporal stationarity assumptions. Exploring the full fMRI signal as a dynamically evolving composition of structured metric 3D spatial patterns is the ultimate goal. Here we have introduced a simple first step which, despite averaging out many effects that would appear in a time and space-varying (non-stationary) version of the analysis has revealed some interesting features of the global signal. Moreover, the fact that even this relatively “blurred” characterization of the spatiotemporal organization of whole-brain resting-state brain activation captures highly significant effects of both gender and mental illness indicates that the 4D frequency domain is a powerful source of information about normative and group-specific brain function.

## Conclusions

The approach we propose is intended to capture information that speaks to the full range of neurophysiological mechanisms governing how information distributes over the brain in space and in time. The salient underlying neurophysiology is happening at molecular, cellular and network scales simultaneously, and we would not expect the same scales to be equally implicated in different neurological disorders. Rather we would hope that knowledge of the relevant neurophysiology in a given condition would generate hypotheses to explain observed high-level spatiotemporal spectral differences, possibly leading to a deeper understanding of how different scales of neurophysiological mechanism interact to produce productive and counterproductive patterns of brain activation. Our analysis shows that abstract (non-located) spatiotemporal organizing principles of brain activation differ across populations of interest, a finding that has implications for the interpretation of FNC results and that we hope will provide a jumping off point for more sophisticated investigations of the ambient spatiotemporal activation dynamics that simultaneously constrain and influence functional networks.

### Conflict of interest statement

The authors declare that the research was conducted in the absence of any commercial or financial relationships that could be construed as a potential conflict of interest.

## Footnotes

^1^A “brain” here is taken to be the hemodynamic signal associated to that brain via a resting state fMRI recording.
